# Area‐Selective Atomic Layer Deposition on Homogeneous Substrate for Next‐Generation Electronic Devices

**DOI:** 10.1002/advs.202414483

**Published:** 2025-04-03

**Authors:** Min‐Jeong Rhee, Byoungjun Won, Young‐Jin Lim, Jeong‐Gyu Song, Sunghyun Kim, Il‐Kwon Oh

**Affiliations:** ^1^ Department of Intelligence Semiconductor Engineering Ajou University Suwon 16499 Republic of Korea; ^2^ Device Research Center Samsung Advanced Institute of Technology Samsung Electronics 130 Samsung‐ro Suwon Gyeonggi‐do 16678 Republic of Korea; ^3^ Department of Electrical and Computer Engineering Ajou University Suwon 16499 Republic of Korea

**Keywords:** area‐selective atomic layer deposition, dielectric, grain boundary, homogeneous surface, leakage currents

## Abstract

Area‐selective atomic layer deposition (AS‐ALD) has focused on controlling the promotion or blocking of precursor molecules on “*heterogeneous*” surfaces comprising different materials. This study proposes a new concept of AS‐ALD on “*homogeneous*” surfaces comprising a single material. In this work, a homogeneous ZrO_2_ substrate is selectively fluorinated using sulfur hexafluoride (SF_6_) gas. The SF_6_ decomposes and incorporates into oxygen vacancies in ZrO_2_, forming F‐terminated surface at grain boundaries (GBs). In the following step, the remaining hydroxyl‐terminated ZrO_2_ areas are blocked by a cyclopentadienyl ligand to prevent aluminum precursor adsorption. Density functional theory and Monte Carlo simulations show that selectively passivated GBs of ZrO_2_ lead to the selective adsorption of ZrCp(NMe_2_)_3_ inhibitors. Selective growth of Al_2_O_3_ along GBs of ZrO_2_ is observed by elemental mapping from transmission electron microscopy. Finally, GB‐selective Al_2_O_3_ increases overalldielectric constant by 15.5% in ZrO_2_/Al_2_O_3_/ZrO_2_ stacks with no increase in leakage currents, showing that the GB‐selective Al_2_O_3_ incorporation suffices to passivate leakage paths through ZrO_2_ GBs. These findings provide fundamental guidelines for performing AS‐ALD on homogeneous surfaces and highlight the potential of this approach for applications in next‐generation electronic devices.

## Graphical abstract

This study demonstrates a self‐aligned passivation approach for grain boundaries on ZrO_2_ surfaces using area‐selective atomic layer deposition (AS‐ALD). Selective deposition of Al_2_O_3_ onto ZrO_2_ grain boundaries effectively enhances dielectric performance while tackling leakage pathways. This method highlights a novel AS‐ALD strategy on homogeneous surfaces for next‐generation electronic devices.

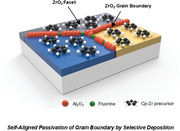

## 1 Introduction

Currently, dynamic random‐access memories (DRAM) are widely used as the main memory in computers and mobile electronic devices owing to their simple design and low cost.^[^ ^1^, ^2^ ^]^ Tetragonal ZrO_2_‐based ZrO_2_/Al_2_O_3_/ZrO_2_ (ZAZ) thin films are used as the dielectric layer in the DRAM cell capacitors of commercial devices.^[^ ^3^ ^]^ To reduce power consumption during DRAM operations, minimizing the leakage currents is essential. In the ZAZ structure, leakage currents typically flow through the grain boundaries (GBs) of ZrO_2_, where leaky paths are passivated by an amorphous Al_2_O_3_ insertion layer. However, this reduces the overall dielectric constant (*k*) value because of the relatively low *k* value (≈9) of Al_2_O_3_ compared to that of tetragonal ZrO_2_ (≈40).^[^ ^4^ ^]^ Consequently, for further down‐scaling, the use of a minimal amount of Al_2_O_3_ is a critical issue to achieve high capacitance in DRAM, as it helps maintain leakage current levels. The continuous downscaling of devices has necessitated only a few cycles of atomic layer deposition (ALD) of Al_2_O_3_ in the high‐volume manufacturing of DRAM devices, ensuring that the Al_2_O_3_ ALD undergoes a limited number of cycles to block the flow of leakage currents between the top and bottom ZrO_2_ layers.

Area‐selective ALD (AS‐ALD) is a bottom‐up film‐growth technique that has recently gained attention for atomic‐scale patterning. ALD growth relies on a self‐limiting surface reaction, allowing proper control of the surface reaction to result in selective interaction between precursor molecules and surface terminations.^[^ ^5^ ^]^ In other words, precise modification of surface terminations through activation/deactivation on *“heterogeneous”* surfaces facilitates the adsorption/desorption of precursor molecules, resulting in the formation of films in desired areas. Previous studies demonstrated that inherent AS‐ALD resulted in selective patterns; for example, W films grew only on hydrogen‐terminated Si surfaces rather than hydroxyl‐terminated Si surfaces.^[^ ^6^, ^7^ ^]^ However, most studies showed failures, such as loss of selectivity after a certain number of cycles, due to the chemical similarities of the hetero‐substrate surfaces. Thus, AS‐ALD on reactive surfaces typically employs inhibitors such as self‐assembled monolayers (SAMs) and small‐molecule inhibitors (SMIs) to maximize selective film thickness.^[^ ^8^ ^]^ Inhibitors such as SAMs and SMIs were selectively deposited on non‐growth areas (NGA) to block deposition during subsequent deposition processes by preventing reactions between the surface and precursors. In contrast to the long carbon chains and incompatible process of SAMs for high‐volume manufacturing, SMIs have attracted significant attention for producing complex 3D structures with high aspect ratios, such as DRAM cell capacitors.^[^ ^9^ ^]^

Performing AS‐ALD on *“homogeneous”* surfaces is more challenging than on “*heterogeneous*” surfaces. A notable example is the work by Chen et al., who focused on inherently selective ALD, suggesting that it involves defect modification within the same material.^[^ ^10^ ^]^ Complementing this, Park et al. conducted research on defect‐selective ALD, specifically targeting GBs in hexagonal ZnO.^[^ ^11^ ^]^ These studies produced significant progress in applications involving homogeneous substrates while highlighting the associated challenges. Unlike heterogeneous substrates, where different materials within a substrate facilitate selective growth owing to their varied chemical properties, homogeneous substrates exhibit similar chemical properties. This similarity had allowed only a few successful cases of selective deposition to date and significant challenges in widening the process window for achieving selective deposition via AS‐ALD.

**Figure 1:**
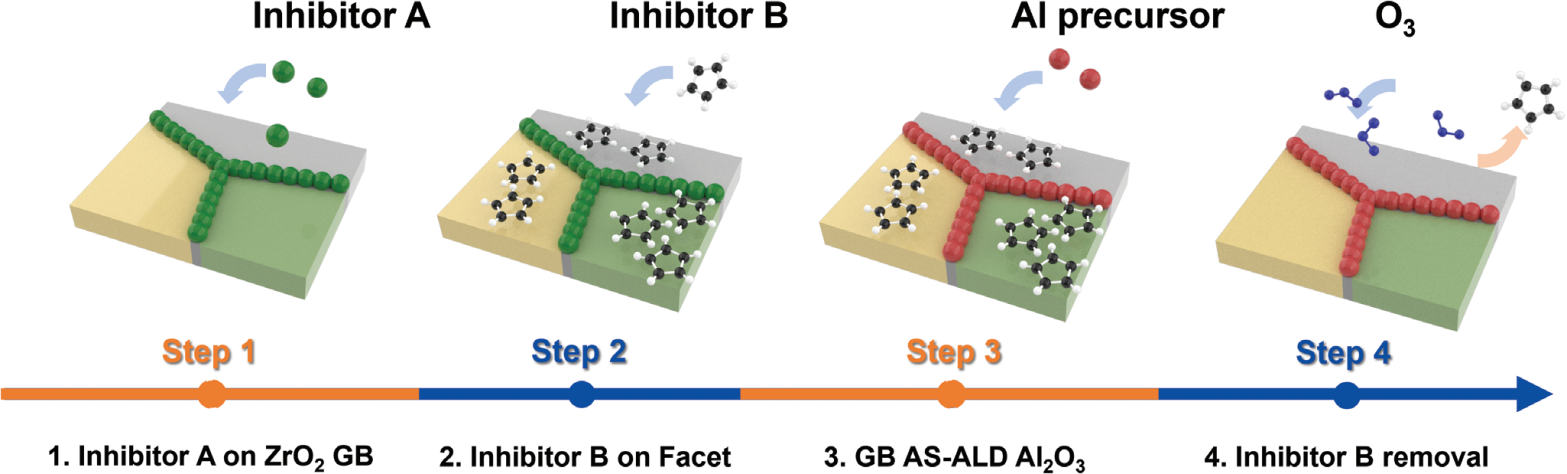
Schematics of the AS‐ALD process for Al_2_O_3_ deposition on ZrO_2_ GBs. Step 1) Selective deposition of *Inhibitor A* exclusively on the GBs of ZrO_2_. Step 2) Selective deposition of *Inhibitor B* only on the ZrO_2_ facet to inhibit Al_2_O_3_ film formation on the facet. Step 3) ALD of Al_2_O_3_ occurs only on the ZrO_2_ GBs coated by *Inhibitor A* (SF_6_), while deposition on the ZrO_2_ facets coated by *Inhibitor B* (ZrCp(NMe_2_)_3_. Step 4) removing inhibitors prior to the deposition of upper ZrO_2_ layers.

Building on the inherent challenges of AS‐ALD on homogeneous substrates, our approach for the selective deposition of Al_2_O_3_ exclusively on ZrO_2_ exemplified a more intricate method based on the differing crystalline structures of the surface (GBs vs facets of ZrO_2_), as depicted in **Figure** **1**. In Step 1, the GBs were selectively passivated with *Inhibitor A* (SF_6_), which affected the ZrO_2_. Step 2 involved passivating the remaining surface area of the ZrO_2_ facet with *Inhibitor B* (ZrCp(NMe_2_)_3_, resulting in cyclopentadienyl (Cp)‐terminated facets. In Step 3, Al_2_O_3_ (red molecules) was deposited on the ZrO_2_ GBs, which were coated with *Inhibitor A*, while deposition on the ZrO_2_ facets was blocked by the Cp‐terminated surface from *Inhibitor B*. Finally, in Step 4, the remaining inhibitors were removed prior to the deposition of the upper ZrO_2_ layers. In contrast to conventional AS‐ALD, which typically employs a single inhibitor, this study incorporated an additional inhibitor step to manage the more complex and challenging process, necessitating precise control at each stage of AS‐ALD.

1. *Inhibitor A*: Previous studies showed that halogen elements can be incorporated into the GBs of metal oxide systems. The incorporation rates depend on molecular size, electronegativity, reactivity, volatility, and steric hindrance.^[^ ^8^, ^12^ ^]^ However, the halogen‐terminated GBs must remain stable upon exposure to the second inhibitor, *Inhibitor B*.
2. *Inhibitor B*: This inhibitor must selectively adsorb onto the ZrO_2_ surface facets without affecting the halogen‐terminated GBs, necessitating precise reactivity windows. In addition, it must be cleanly removed without leaving impurities that could degrade device performance. Heteroleptic Zr precursors, such as tris(dimethylamino)Cp zirconium (ZAC; ZrCp(NMe_2_)_3_), are potential candidates due to their intermediate reactivity from the alkyl amide ligands and low reactivity to block Al precursor adsorption in the subsequent step from the Cp ligand. The Zr─Cp bond remains stable against H_2_O,^[^ ^13^ ^]^ which is used in the subsequent ALD of Al_2_O_3_. In addition, Cp‐type Zr precursors have been widely used for ZrO_2_ ALD, and the remaining Cp ligands can effectively removed by O_3_ treatment, converting them into ZrO_2_ films at the end of AS‐ALD.
3. Al precursor: In our previous work,^[^ ^14^, ^15^ ^]^ the size and Lewis acidity of the precursors were critical for achieving high selectivity. We compared the adsorption and blocking properties of two Al precursors, trimethylaluminum (TMA, AlMe_3_) and dimethyl isopropyl aluminum (DMAI, AlMe_2_iPrO), based on previous reports.^[^ ^14^, ^15^, ^16^ ^]^

The reaction mechanisms were elucidated through a correlative study of density functional theory (DFT) calculations, Monte Carlo (MC) simulations, and experimental observations. The selectivity and chemical composition of the ZrO_2_ surface were investigated using X‐ray photoelectron spectroscopy (XPS), and its crystallinity was investigated using the X‐ray diffraction (XRD). The selective adsorption of chemical species and the growth of Al_2_O_3_ on ZrO_2_ were visually observed using transmission electron microscopy (TEM) with energy‐dispersive X‐ray spectrometer (EDS) and electron energy loss spectroscopy (EELS). Finally, we evaluated the device performance of the DRAM, configured as a metal‐insulator‐metal (MIM) capacitor incorporating AS‐ALD Al_2_O_3_ in the ZAZ structure. This thorough study of AS‐ALD on *“homogeneous”* surfaces, integrating both experimental and theoretical methods, will enable a deeper understanding of the fundamental role that inhibitor/precursor selection plays in the development of next‐generation semiconductor devices.

## 2 Results and Discussion

**Figure 2:**
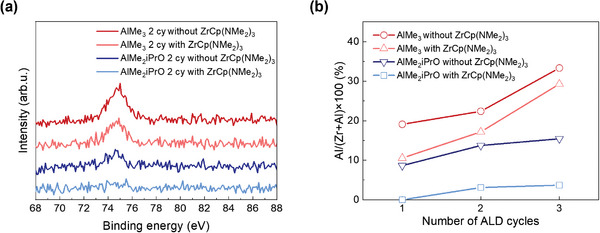
a) Al 2p peak in XPS data after two cycles of Al_2_O_3_ ALD on ZrO_2_. b) Relative Al concentration ((amount of Al)/(amount of Zr+amount of Al) x 100) when using AlMe_3_ and AlMe_2_iPrO on ZrO_2_ as with/without ZrCp(NMe_2_)_3_.

Before investigating the passivation effects of *Inhibitor A* and *Inhibitor B*, we examined the impact of blocking Al precursors in advance. The XPS core‐level spectra in **Figure** **2**a illustrate the Al 2p levels for two cycles of Al_2_O_3_ ALD on ZrO_2_, both with and without ZrCp(NMe_2_)_3_ coating (*Inhibitor B*). The reduced Al in the case of ZrCp(NMe_2_)_3_ exposure indicates that the ZrCp(NMe_2_)_3_ precursor exposure effectively blocks the adsorption of AlMe_3_ and AlMe_2_iPrO molecules on the surface. Notably, AlMe_2_iPrO demonstrated the difference in a lower adsorption level compared to AlMe_3_, suggesting better blocking efficiency. The relative Al amount ratios from the XPS results are presented in Figure 2b, with raw data shown in Figure S1 (Supporting Information). In both cases—whether exposed to ZrCp(NMe_2_)_3_ or not—an increase in Al peak intensity was observed as the number of ALD cycles increased from one to three. Intriguingly, in all cases, the sample treated with ZrCp(NMe_2_)_3_ exhibited reduced Al deposition compared to the sample without ZrCp(NMe_2_)_3_ treatment. This observation substantiates the effectiveness of *Inhibitor B* in blocking Al_2_O_3_ deposition, underscoring its potential as a selective agent for AS‐ALD.

Recent studies conducted by our group^[^ ^14^, ^15^ ^]^ and Merkx et al.^[^ ^12^ ^]^ highlighted the importance of precursor selection for achieving selectivity in AS‐ALD processes. These previous investigations led us to select two distinct Al precursors, AlMe_3_ and AlMe_2_iPrO, based on several key criteria: compatibility in the high‐volume manufacturing of semiconductors, convenience of a liquid form for ease of processing and reduced contamination risks, and a composition devoid of halide ligands to minimize contamination. The XPS core‐level spectra showed that AlMe_2_iPrO exhibited inherently lower peak intensities, suggesting a naturally reduced deposition rate compared to AlMe_3_. Notably, during the initial deposition cycles, AlMe_2_iPrO with ZrCp(NMe_2_)_3_, *Inhibitor B* exhibited almost no Al deposition, indicating its high selectivity for AlMe_2_iPrO. It demonstrates that using AlMe_2_iPrO with ZrCp(NMe_2_)_3_ is a more targeted approach for decreasing Al_2_O_3_ amount on the ZrO_2_ facet during ALD.

**Figure 3:**
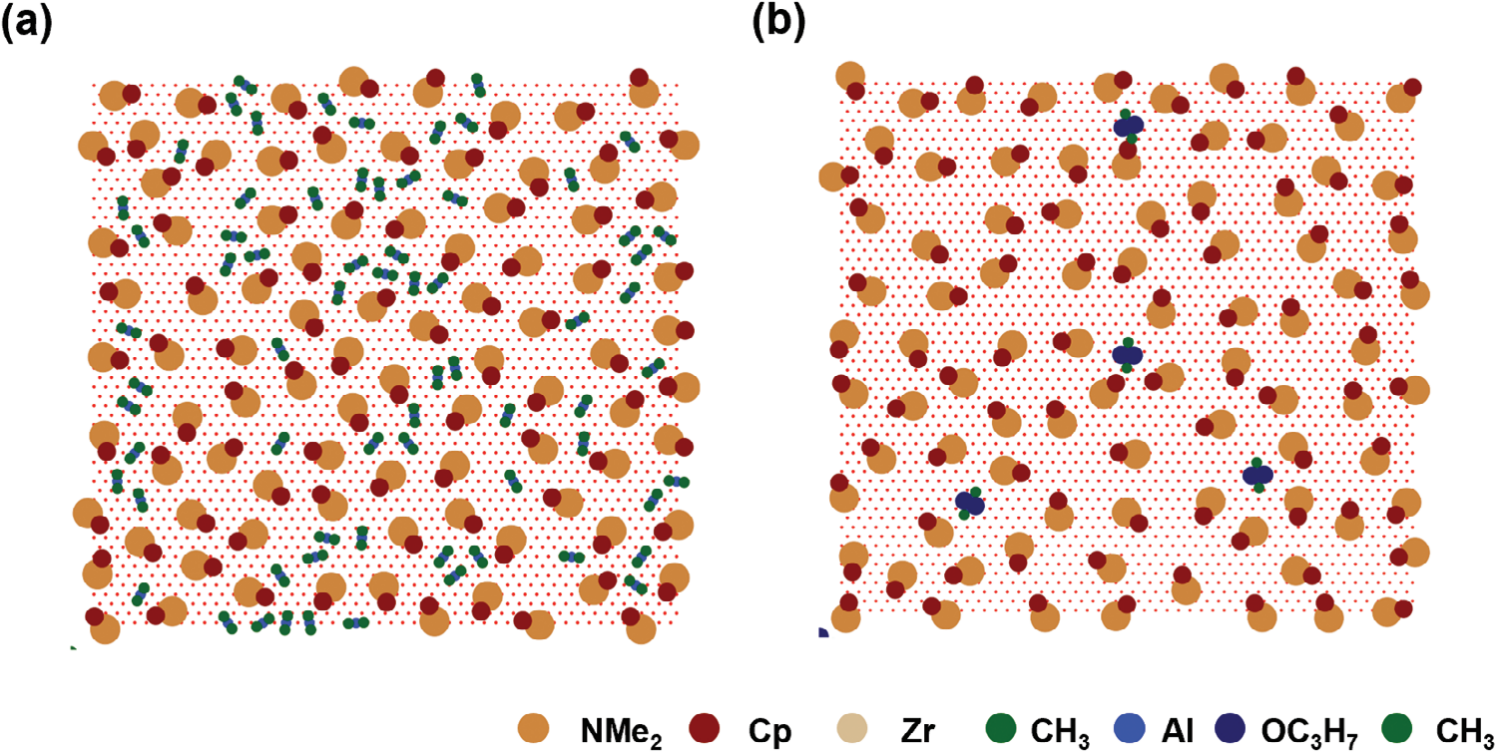
MC simulation results a) AlMe_3_ on ZrCp(NMe_2_)_3_ result b) AlMe_2_iPrO on ZrCp(NMe_2_)_3_ result when AlMe_2_iPrO acts as a dimer.

Having observed a reduced Al ratio with AlMe_2_iPrO as the precursor, as depicted in Figure 2, we explored the mechanistic aspects of this phenomenon using MC simulations, as shown in **Figure** **3**. Choi et al. showed that during ALD reactions using ZrCp(NMe_2_)_3_, the most favorable configuration involved one NMe_2_ ligand and one Cp ligand, as the remaining two NMe_2_ ligands reacted with the hydroxyl‐terminated surface.^[^ ^13^ ^]^ In addition, as shown in Figure S2 (Supporting Information), the reaction energies of the amido and Cp ligands with AlMe_3_ and AlMe_2_iPrO indicated that reactions between the remaining ligands and Al precursors were not energetically favorable. Therefore, during the MC simulations, we considered independent molecules that did not undergo any chemical reactions. Also, according to the previous study by H. B. R. Lee et al., AlMe_3_ molecules predominantly existed as monomers owing to their lower dimerization energy, while AlMe_2_iPrO molecules exhibited a tendency to dimerize, given their higher dimerization energy at 200 °C.^[^ ^16^ ^]^ ZrCp(NMe_2_)_3_ molecules were sprayed first onto the substrate, resulting in saturated coverage up to a certain level (22.04%–22.30%). Upon exposing monomer AlMe_3_ molecules to the Cp‐terminated surface, the Al coverage was 8.69%, whereas the dimer AlMe_2_iPrO had an extremely low value of 0.88%. This indicated that differences in the precursor size augmented steric hindrance, thereby improving the blocking effect. Specifically, AlMe_2_iPrO predominantly existed in the form of dimers, which increased in size and resulted in a significantly lower coverage of 0.88%. The low coverage of AlMe_2_iPrO, reflected in the reduced Al ratio, aligned with the experimental findings presented in Figure 2. When comparing both Al precursors in their monomer forms, the AlMe_2_iPrO case exhibited a lower Al coverage (7.01%) compared to the AlMe_3_ case (8.69%) (see Figure S3, Supporting Information). These results, along with the enhanced blocking efficiency of AlMe_2_iPrO, led to its selection as the precursor for subsequent experiments. Moreover, considering the selective window and effectiveness of blocking the leakage current of AlMe_2_iPrO, Al_2_O_3_ was conducted in 2 cycles.

**Figure 4:**
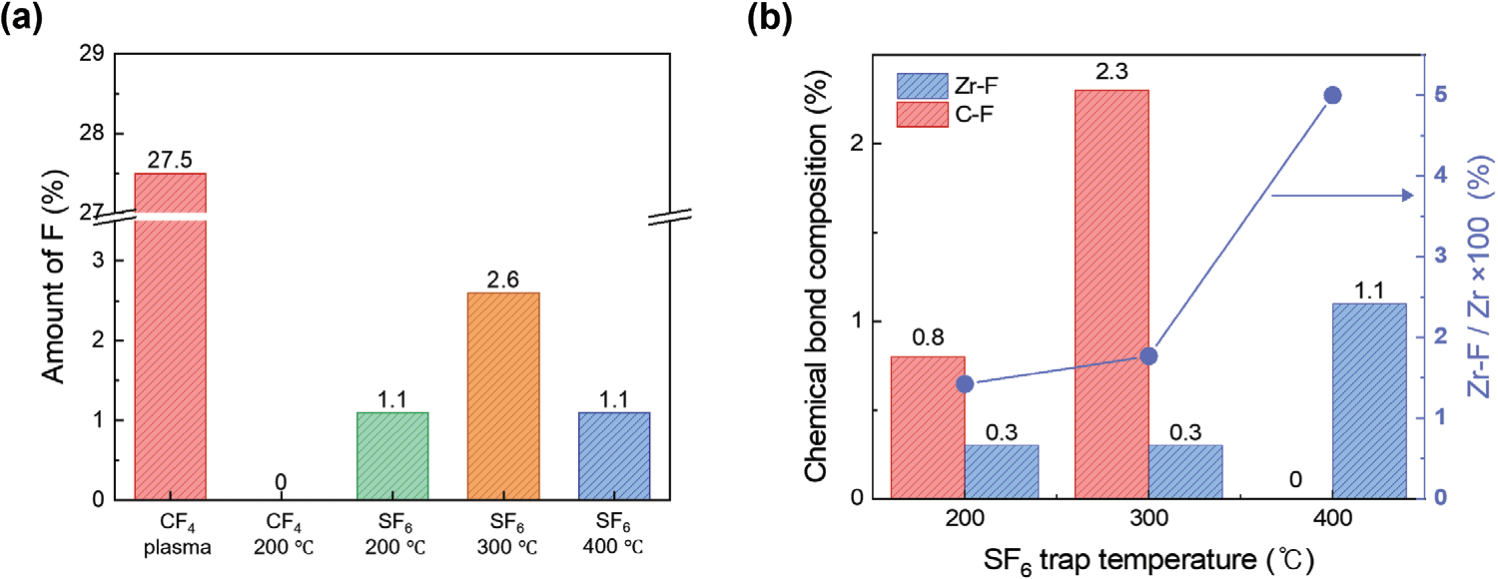
Chemical composition of the ZrO_2_ surface after halogenation. a) Amount of fluorine detected via XPS following CF_4_ plasma treatment at 25 °C, CF_4_ gas exposure at 200 °C, SF_6_ gas exposure at 200, 300, and 400 °C on 10 nm ZrO_2._ b) Composition of Zr─F and C─F bonds, along with the Zr−F/Zr ratio, detected via XPS after SF_6_ gas exposure experiments conducted at 200, 300, and 400 °C on 10 nm ZrO_2_.

To study the effect of *Inhibitor A*, experiments were conducted by applying various gases or plasma treatments to identify the traps on the ZrO_2_ surface, and the presence of fluorescence was subsequently detected using XPS. A grain size in 3 nm thick ZrO_2_ films, being relatively small, would make it challenging to detect F elements accurately; therefore, a thickness of 10 nm was considered. As indicated in **Figure** **4**a, the amount of F after the CF_4_ plasma treatment was relatively large. As our study focused only on trapping GBs on the surface, lower levels of *Inhibitor A* adsorption were required. F was not detected on the surface when surface treatments were performed using CF_4_ gas without plasma. To implement more effective gas trapping, we used an F‐containing material with a decomposition temperature lower than that of CF_4_. Further analysis revealed that SF_6_ had a lower decomposition temperature than CF_4_,^[^ ^17^ ^]^ suggesting greater F incorporation on the surface of the ZrO_2_ layers. Experiments conducted at 200, 300, and 400 °C using SF_6_ gas showed surface F amounts ranging from 1.1% to 2.6%, as shown in Figure 4a. The lower‐temperature decomposition of SF_6_^[^ ^17^ ^]^ facilitated improved controllability and effectiveness of trapping, supporting our hypothesis that SF_6_ is a more efficient gas for trapping. Furthermore, as shown in Figure S4 (Supporting Information), the XPS data of the F 1s peak analysis revealed that at 200 and 300 °C, both C─F and Zr─F bonds were detected on the surface. In contrast, at 400 °C, only Zr─F bonds were detected, without the presence of C─F bonds. Figure 4b summarizes the composition of Zr−F and C−F, showing that the Zr−F ratio is highest at 400 °C. The bond of carbon to fluorine is considered from a reaction with surface carbon, generated between the ALD process and the trap‐annealing process^[^ ^18^ ^]^ It remains at temperatures of 200∼300 °C, but that surface carbon decomposes and/or evaporates at temperatures above 400 °C.^[^ ^18^ ^]^ Therefore, in this study, we optimized the SF_6_ gas trap by conducting tests at 400 °C, where no C─F bonds were formed.

**Figure 5:**
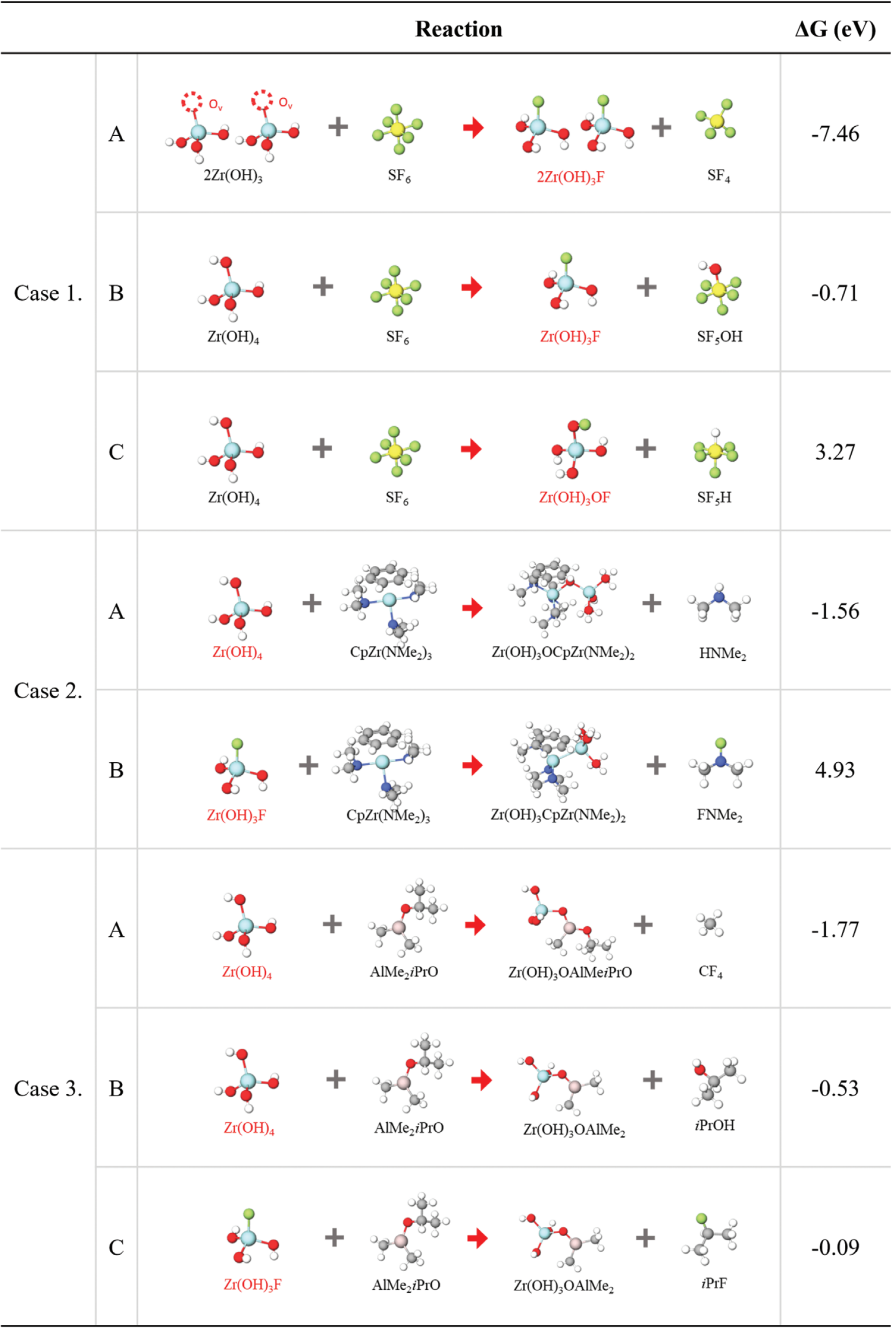
Changes in O 1s peak in XPS according to different treatments on Al_2_O_3_ deposited on ZrO_2_. a) Al_2_O_3_ on ZrO_2_, b) Al_2_O_3_ on ZrO_2_ with Cp treatment, c) Al_2_O_3_ on ZrO_2_ with F treatment, and d) Al_2_O_3_ on ZrO_2_ with F and Cp treatment. The percentages indicate the relative contributions of the subpeak in the O 1s spectrum.

The chemical composition of the surface was investigated experimentally. **Figure** **5** shows the XPS analysis of the O 1s core level, with and without *Inhibitor A* (F trap) and *Inhibitor B* (Cp); the four samples were the control samples of ZrO_2_/Al_2_O_3_/ZrO_2_ (denoted as ZAZ), ZrO_2_/Al_2_O_3_/ZrO_2_ with *Inhibitor B* (denoted as ZAZ+Cp), ZrO_2_/Al_2_O_3_/ZrO_2_ with *Inhibitor A* (denoted as ZAZ+F), and ZrO_2_/Al_2_O_3_/ZrO_2_ with *Inhibitors A* and *B* (denoted as ZAZ+F+Cp). All the peaks were calibrated with the C─C bond set at 284.8 eV,^[^ ^19^ ^]^ while the main Zr−O peak was fixed at 530.0 eV.^[^ ^20^ ^]^ Subpeaks were observed near 532 eV, corresponding to defective bonds. The peak at 531.2–531.3 eV was reported as an oxygen vacancy (V_O_) peak,^[^ ^21^, ^22^ ^]^ whereas the peak at 531.3–532.2 eV was associated with the OH peak.^[^ ^20^, ^23^ ^]^ Regarding the deconvolution of the OH peak and O_v_ peak in a sub‐oxide peak, we could not assure since it is difficult to accurately separate these peaks due to their close proximity (only ≈1 eV apart). For this reason, we have grouped them under the sub‐oxide peak in Figure 5 to avoid confusion. Comparing Figure 5a,b with Figure 5c,d, respectively, the subpeak was found to decrease upon the addition of F‐trapping *Inhibitor A*. Furthermore, when comparing Figure 5a,c with Figure 5b,d, respectively, a decrease in the subpeak was observed. These experimental results will be further explained in the following section through DFT analysis.

**Table** **1** presents the elemental chemical compositions of Al, F, Zr, and O obtained from the samples analyzed in Figure 5. The samples were divided as follows: the traditional ZrO_2_/Al_2_O_3_/ZrO_2_ structure (ZAZ), ZrO_2_/Al_2_O_3_/ZrO_2_ with *InhibitorB* (ZAZ+Cp), ZrO_2_/Al_2_O_3_/ZrO_2_ with *Inhibitor A* (ZAZ+F), and ZrO_2_/Al_2_O_3_/ZrO_2_ with both *Inhibitors A* and *B* (ZAZ+F+Cp). The results are presented in Figure S5 (Supporting Information). The ZAZ sample, which represented the pristine deposition of Al_2_O_3_ on ZrO_2_ without any inhibitor, exhibited the highest Al ratio. The reduction in the Al ratio from ZAZ to ZAZ+Cp was discussed in Figure 2. ZAZ+F, in which *Inhibitor A* was treated, showed a lower Al atomic concentration of 1.5%, which decreased from 15.8% for ZAZ. Notably, the presence of *Inhibitor A* leads to a diminished reactivity of the surface toward Al_2_O_3_ deposition in ZAZ+Cp sample. When comparing ZAZ+F+Cp with ZAZ+Cp, the Al ratio was found to be lower in the ZAZ+Cp case, indicating a stronger inhibitory effect of Cp alone compared to the co‐presence of F and Cp. This supported our hypothesis that in the case of ZAZ+F+Cp, *Inhibitor A* mainly adsorbed onto V_O_, and *Inhibitor B* subsequently attached to the remaining OH groups. These OH groups, which terminate ZrO_2_ facets, are formed during O_3_‐based ALD processes due to interactions between reactive oxygen species and the ZrO_2_ surface.^[^ ^24^ ^]^ Additionally, oxygen vacancies are formed during grain formation through post‐deposition annealing, as structural instabilities and lattice mismatches at grain boundaries.^[^ ^25^ ^]^ As a result, both the F and Cp groups remained on the surface. However, for ZAZ+Cp, without the initial presence of F, *Inhibitor B* covered the surface more effectively, blocking the V_O_ and OH groups. This extensive coverage by Cp groups, which are less reactive than OH or F, led to even lesser interaction with AlMe_2_iPrO, resulting in a further reduction in the Al ratio. In this scenario lacking highly reactive OH and presenting less reactive Cp groups, the Al precursors would mainly react with the F groups, reducing the Al ratio. A noteworthy observation was the absence of F in ZAZ+F+Cp. This suggested a potential reaction mechanism in which F was eliminated from the surface during Al_2_O_3_ deposition.

**Table 1:** Chemical properties using XPS data. a) Al_2_O_3_ on ZrO_2_ result, b) Al_2_O_3_ on ZrO_2_ with Cp result, c) Al_2_O_3_ on ZrO_2_ with F treatment, and d) Al_2_O_3_ on ZrO_2_ with F treatment and Cp result. The Al ratio is calculated as (Al/(Zr+Al) × 100%).

	1) ZAZ	2) ZAZ+Cp	3) ZAZ+F	4) ZAZ+F+Cp
Al	4.8	0.9	1.5	1.0
F	‐	‐	‐	‐
Zr	25.5	28.2	20.1	19.6
O	69.7	70.9	77.5	79.4
Al ratio	15.8%	3.1%	6.9%	4.9%

**Figure 6:**
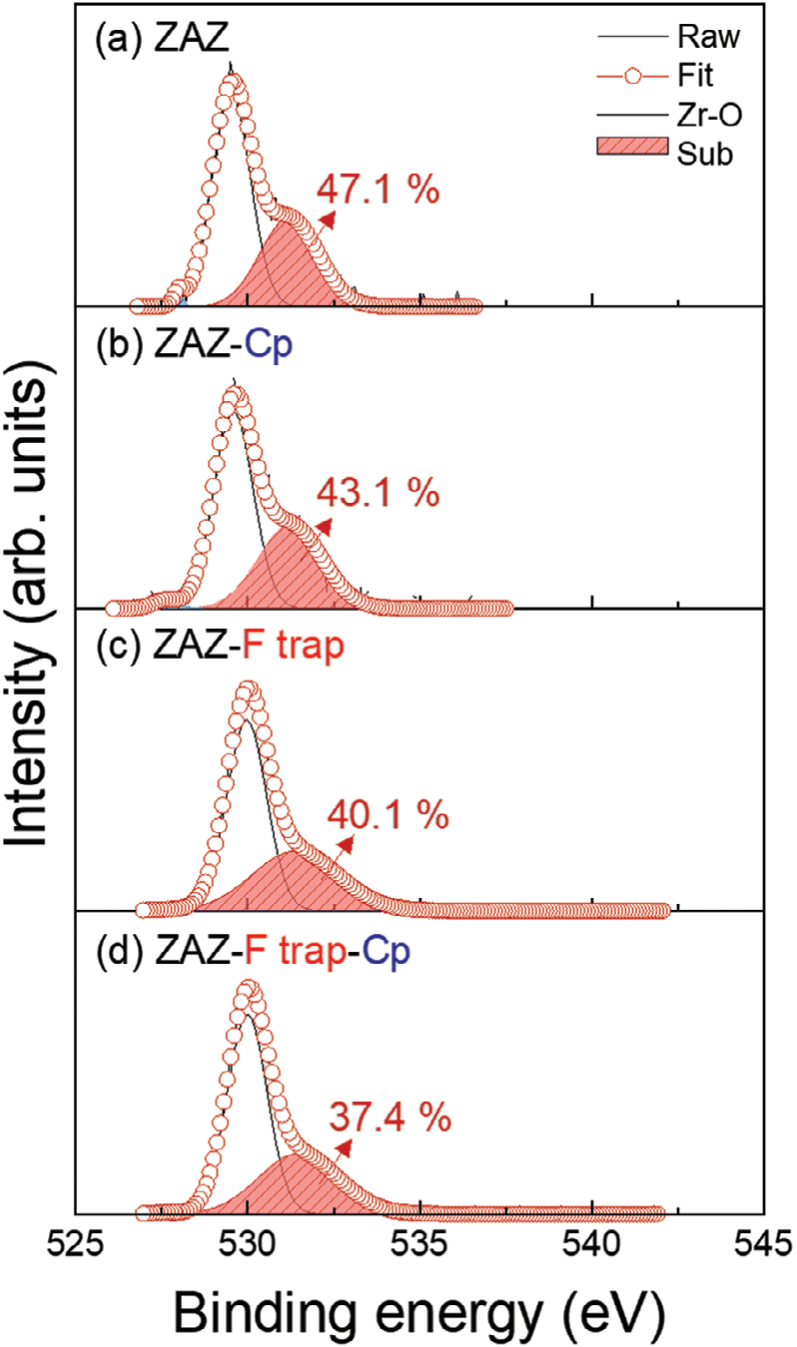
DFT‐calculated Gibbs free energy (ΔG) for various reactions (Case 1.) Adsorption of SF₆ molecules on ZrO₂ surfaces: Zr─F bond formation at oxygen vacancy sites (Case 1‐A), Zr−F formation at Zr−OH sites (Case 1‐B), and Zr−OF formation at Zr‐OH sites (Case 1‐C). (Case 2.) Adsorption of ZrCp(NMe_2_)_3_ molecules: adsorption on Zr−OH sites (Case 2‐A) and adsorption on Zr−F sites (Case 2‐B). (Case 3.) Adsorption of AlMe_2_iPrO molecules: reaction of *i*PrO ligand with Zr−OH sites (Case 3‐A), reaction of ligand with Zr−OH sites (Case 3‐B), and reaction with Zr‐F sites (Case 3‐C). The elements in the figure are represented as follows: Zr is cyan, O is red, H is white, S is yellow, F is green, N is blue, C is gray, and Al is pink.

To elucidate the reaction mechanism in AS‐ALD, we calculated the Gibbs free energy (Δ*G*) of the adsorption of SF_6_ molecules on ZrO_2_ surfaces (Case 1), with two different terminations of Zr—oxygen vacancy (Case 1‐A) and OH (Case 1‐B and Case 1‐C) by DFT, as shown in **Figure** **6**. The most favorable reaction pathway turned out to be the reaction with oxygen vacancies (−7.46 eV), whereas the reaction with hydroxyl was less favorable (−0.71 and 3.27 eV). Because it has been known that GBs are 1D linear connections of oxygen vacancies,^[^ ^26^, ^27^ ^]^ the reaction of SF_6_ with ZrO_2_ is more likely to occur at the GB. However, fluorine not only reacts with oxygen vacancies but also with hydroxyl groups, as shown in Figure 6. This dual reactivity likely explains the reduced Al peak observed in the XPS results for the ZAZ+F sample, as shown in Table 1, where fluorine passivation inhibits Al adsorption. Another possible explanation for this reduction may partly be attributed to the presence of amorphous regions in the ZrO_2_ film, as will be discussed in the TEM analysis. In such regions, fluorine may interact with additional reactive sites, further enhancing the suppression of Al_2_O_3_ deposition. This observation underscores the limitations of DFT calculations in fully capturing the structural heterogeneity of the ZrO_2_ surface. We also calculated the ΔG of the adsorption of ZrCp(NMe_2_)_3_ molecules (Case 2) by DFT, as reactions with two terminations—Zr−OH (Case 2‐A) or Zr−F (Case 2‐B). The Δ*G* for Zr−OH was −1.56 eV, much lower than that for Zr−F, 4.93 eV. This indicated that most of the ZrCp(NMe_2_)_3_ precursors were adsorbed on Zr−OH sites rather than on the F‐terminated surface. Energetically, SF_6_ molecules were favorably adsorbed on the oxygen vacancies, mainly on ZrO_2_ GBs, rather than the facet mainly comprising Zr−OH sites. The homogeneous surface transformed into a heterogeneous surface comprising Zr−OH and Zr−F sites. Most ZrCp(NMe_2_)_3_ precursors tended to adsorb on the Zr−OH sites rather than on the Zr−F sites, resulting in a surface that featured Cp‐terminated ZrO_2_ regions due to reactions, along with F‐terminated Zr regions from blocking. Finally, we performed calculations of the adsorption of the AlMe_2_iPrO precursors (Case 3) on Zr−OH (Case 3‐A and Case 3‐B) and Zr−F (Case 3‐C). For the Zr−OH sites, two reaction pathways were considered, resulting in energies of −1.77 eV for reacting with the −CH_3_ ligand and −0.53 eV for *i*PrO, showing that both pathways were thermodynamically favorable. However, since the ZrCp(NMe_2_)_3_ precursors had already occupied the Zr−OH sites as terminating Cp ligands on the surface, many Zr−OH sites were not available to react with the Al precursors. In contrast, although the reaction of Al precursors with Zr−F did not show a very high value (−0.09 eV), it still had a chance for the reaction because the remaining area had already been packed with the Cp ligand (no reaction with AlMe_2_iPrO precursor, as shown in Figure S2, Supporting Information). In addition, *Inhibitor B* interfered with the adsorption of AlMe_2_iPrO, as shown in Figure 2. Furthermore, F was mainly incorporated into the oxygen vacancies on the ZrO_2_ surface (mainly along the GBs), forming Zr–F sites. The ZrCp(NMe_2_)_3_ molecules mainly reacted with Zr−OH, and not with Zr−F. Thus, the AlMe_2_iPrO Al precursors would react mainly along the GBs of ZrO_2_ rather than remaining on the ZrO_2_ facets with passivated Cp ligands.

**Figure 7:**
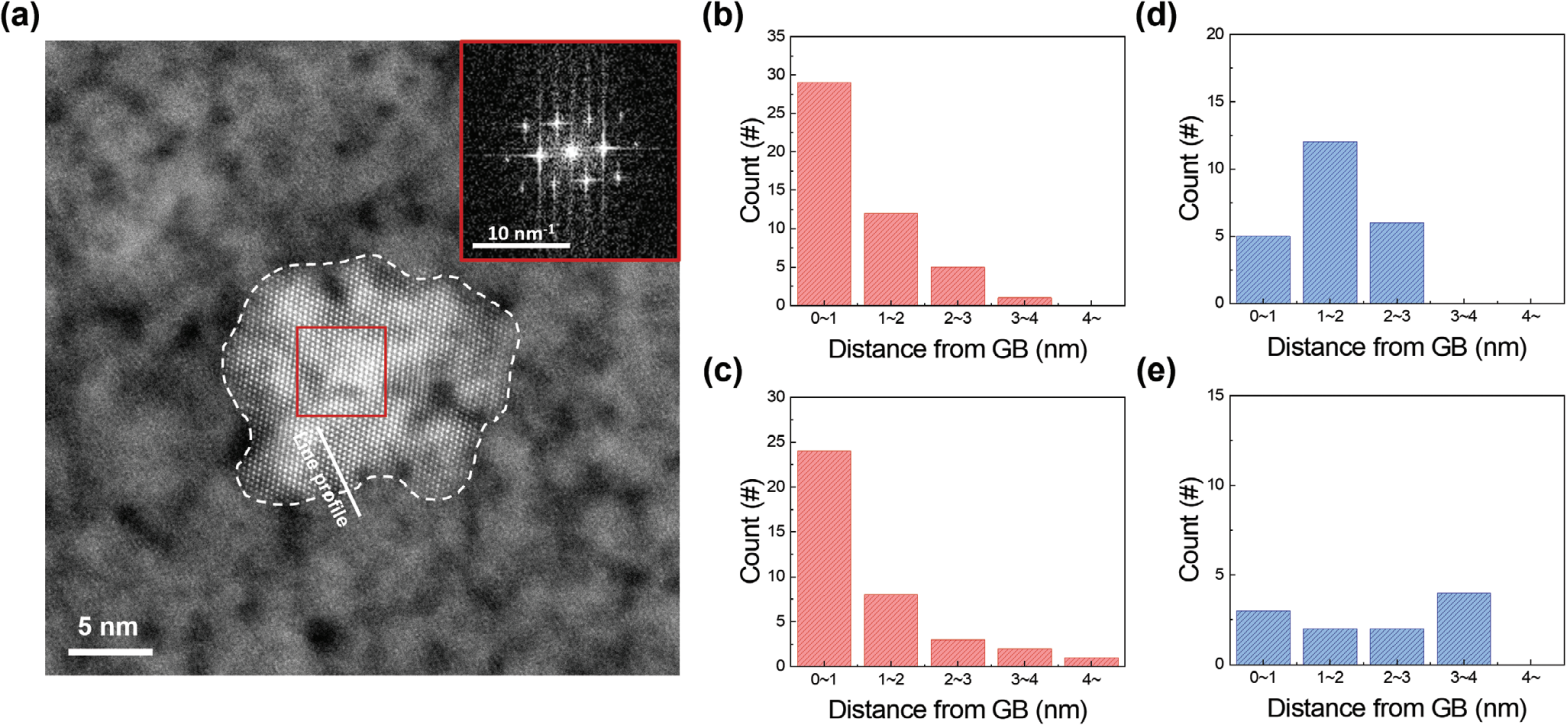
a) Top‐view TEM image of ZrO_2_, along with its corresponding FFT image. b) Statistical analysis of the highest Al intensity and its distance from the GB using EDS line profiles for AS‐ALD applied samples. c) Statistical analysis of the highest Al intensity distribution using EELS line profiles for AS‐ALD applied samples. d) Statistical analysis of the highest Al intensity and its distance from the GB using EDS line profiles for general ALD comparison samples. e) Statistical analysis of the highest Al intensity distribution using EELS line profiles for general ALD comparison samples.

We statistically observe the Al distribution on the ZrO_2_ surface after AS‐ALD through TEM EDS and EELS. **Figure** **7**a shows a grain of ∼20 nm ZrO_2_ in diameter. The corresponding fast Fourier transform (FFT) patterns in the inset of Figure 7a confirm the crystallinity of the grains of ZrO_2_. This provides a solid basis for further EDS and EELS analyses to investigate the elemental distributions, particularly the Al concentration near the GBs. However, although we attempted to directly detect F signals after SF_6_ treatment using EDS and EELS, it was not successful due to technical limitations such as the low F concentration and signal interference in EDS and EELS measurement. Similarly, obtaining reliable EDS and EELS mapping images for Al proved challenging due to the inherently low signal intensity and noise interference from the thin Al_2_O_3_ layer, so we utilized line profile analyses instead, which provide higher sensitivity and are more suitable for quantitative analysis of weak signals. We performed elemental line‐scanning of both EDS and EELS analyses shown in Figure S7c,d (Supporting Information) respectively, along the white lines. In both cases, the highest Al peak was observed near the GBs. Given that the amount of Al_2_O_3_ deposited was extremely small, statistical analysis was performed to ensure the reliability of our findings, as shown in Figure 7b,c. The statistical analysis in Figure 7b using EDS reveals that 61.70% of the highest Al peaks are located within 1 nm from the GBs. Similarly, the EELS analysis in Figure 7c shows that 63.16% of the Al peaks are concentrated within 1 nm from the GBs. This statistical verification further validates the preferential formation of Al_2_O_3_ along the GBs rather than on the ZrO_2_ facets. In contrast, Figure 7d,e present line‐scanning results of EDS and EELS, respectively, for the control sample of non‐selective ALD forming uniform Al_2_O_3_ layers on ZrO_2_ from Figure S7g,h (Supporting Information), respectively. These results show that 21.74% and 27.27% of the Al peaks are located within 1 nm of the GBs, respectively, which are similar to the area out of GBs. It indicates that no notable selective growth of Al_2_O_3_ occurs along the GBs during ALD without inhibitors.

**Figure 8:**
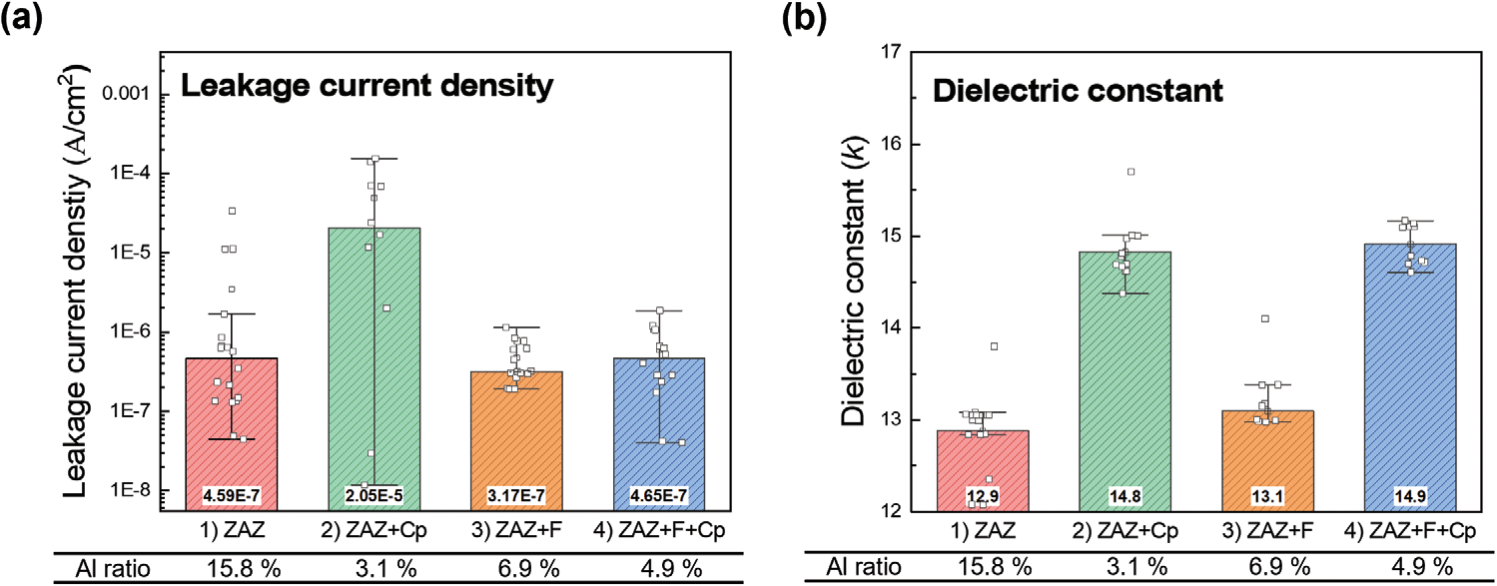
Electrical properties of MIM device, where the mean values are shown inside the bars, are calculated after removing outliers using the 1.5 interquartile range criteria. a) Leakage current density of ZAZ, ZAZ+Cp, ZAZ+F, and ZAZ+F+Cp device separately. b) The dielectric constant of ZAZ, ZAZ+Cp, ZAZ+F, and ZAZ+F+Cp devices separately.

Finally, we study the effect of the selective process and evaluate electrical properties by MIM capacitors with ZAZ, ZAZ+Cp, ZAZ+F, and ZAZ+F+Cp. Leakage current density at 1 MV cm^−1^ and dielectric constants from all devices were measured eight times, and the results are presented as distributions (dots) and means (bars and labels), as shown in **Figure** **8**. The outliers in the data are removed using the 1.5 interquartile range (IQR) criteria to ensure the accuracy and reliability of the results. The electrical properties are influenced by the conditions of the top and bottom ZrO_2_ films, where smaller grain sizes increase grain boundary density, leading to more leakage paths and higher leakage currents.^[^ ^28^ ^]^ To exclude these effects and focus solely on the impact of AS‐ALD, we ensured identical ZrO_2_ process conditions for both layers When comparing ZAZ and ZAZ+Cp (with Cp treatment), both the leakage current and dielectric constant increase. By correlating the amounts of Al_2_O_3_ in Table 1, it is evident that the ZAZ sample exhibits an Al ratio of 15.8%, whereas ZAZ+Cp presents a significantly reduced Al ratio of 3.1%. A reduction of Al amount increases dielectric constant; however, it also increases leakage currents because of the failure of electron insulating due to the absence of Al_2_O_3_ layers. In contrast, when comparing ZAZ and ZAZ+F, both parameters remain at almost similar levels. Since the amount of Al decreased from 15.8% to 6.9%, an increase in the dielectric constant would be expected. However, the incorporated fluorine as an impurity might negatively affect the dielectric constant,^[^ ^29^ ^]^ thereby offsetting the potential increase and resulting in a similar level. In contrast, the fluorine could passivate defects inside ZrO_2_ films resulting in the reduction of leakage currents.^[^ ^30^, ^31^, ^32^, ^33^, ^34^ ^]^ Thus, it might compensate the role of Al_2_O_3_ layers. Finally, when comparing ZAZ and ZAZ+F+Cp, the leakage remains at similar levels, but the *k* value markedly increases by 15.5%. Thus, after AS‐ALD, although the amount of Al_2_O_3_ is reduced, it mainly exists along the GBs enabling the blocking of electron conduction, similar to the uniform Al_2_O_3_ layers (a ZAZ sample). The reduced Al content increases the dielectric constant. This contributes to an overall increase in the dielectric constant while maintaining the level of leakage current. These findings provide valuable insights for the design and optimization of surface modification strategies for next‐generation electronic devices.

## 3 Conclusion

In this study, we successfully demonstrate the AS‐ALD on *homogeneous* ZrO_2_ substrate and fundamentally study the surface chemistry. By utilizing a dual‐inhibitor approach, with SF_6_ as *Inhibitor A* for selective fluorination of GBs and ZrCp(NMe_2_)_3_ as *Inhibitor B* for blocking Al precursor adsorption on ZrO_2_ facets, we achieve selective deposition of Al_2_O_3_. Through DFT calculations and experimental observation, we identify that SF_6_ preferentially adsorbs at oxygen vacancies along the GBs, while ZrCp(NMe_2_)_3_ blocks Al precursor adsorption on ZrO_2_ facets. Furthermore, the employment of ZrCp(NMe_2_)_3_ reduces Al content from 15.8% to 4.9%, demonstrating the effective blocking of Al precursors. Consequently, dual‐inhibitor selective deposition (ZAZ+F+Cp) improves the dielectric constant by 15.5%, while maintaining the leakage current density compared to conventional ZAZ, efficiently managing the leakage path and improving the material properties, highlighting the potential of AS‐ALD in electronic devices. Consequently, the selective deposition improves the dielectric constant by 15.5%, while maintaining leakage current density, highlighting the potential for AS‐ALD in electronic devices by efficiently managing leakage paths and enhancing material properties. These findings offer valuable insights into the design and optimization of surface modification strategies for next‐generation electronic devices.

## 4 Experimental Section

ZrO_2_ thin films were deposited on TiN wafers (previously deposited by ALD on SiO_2_ substrate) at a substrate temperature (T_s_) of 250 °C using a commercial ALD chamber (Atomic Premium, CN1 Co., Ltd.)., ZrCp(NMe_2_)_3_ (Humist Co., Ltd.) was used as the ZrO_2_ precursor, which also served as *Inhibitor B*. O_3_ was used as the reactant, and the flow rate was maintained at 180 sccm. The ZrCp(NMe_2_)_3_ molecules, contained in a stainless‐steel bubbler, were vaporized at 75 °C to achieve a sufficient vapor pressure (∼25 mTorr), and the delivery lines were heated to 90 °C to prevent precursor condensation. Al_2_O_3_ thin films were deposited at the same T_s_ (250 °C) in the same ALD chamber using AlMe_3_ (Humist Co., Ltd.) and AlMe_2_iPrO (Humist Co., Ltd.). Instead of O_3_, less reactive H_2_O was used as the reactant for Al_2_O_3_ deposition to prevent damaging or oxidizing the pre‐adsorbed Zr inhibitor, *Inhibitor B*. The AlMe_3_ precursor was vaporized at room temperature, while AlMe_2_iPrO was vaporized at 55 °C, and their delivery lines were heated to 50 and 70 °C, respectively, to avoid condensation. Ar carrier gas was supplied at 100 sccm to transport the precursor vapors to the reaction chamber, with the same Ar flow rate used for purging excess gas molecules and byproducts between precursor and reactant exposures. All films deposited using ZrCp(NMe_2_)_3_, AlMe_3_, and AlMe_2_iPrO exhibited typical ALD growth characteristics, as shown in Figure S8 (Supporting Information). XPS analysis (Thermo Fisher Scientific, NEXSA) showed negligible impurity levels in the ZrO_2_ and Al_2_O_3_ films, with impurity levels below the XPS detection limit (Figure S9 and Table S1, Supporting Information). The surface C 1s peak (284.8 eV) was used as a reference to calibrate the measured core levels.

The bottom ZrO_2_ layers were prepared in two distinct ways to fabricate crystalline and amorphous ZrO_2_ thin films. XRD (SmartLab, Rigaku) was used to investigate the crystalline structures of these ZrO_2_ layers (Figure S10, Supporting Information). A thickness of ∼3 nm of ZrO_2_ layers was achieved on TiN substrates over 27 cycles. The as‐deposited sample exhibited an amorphous phase. To create crystallized ZrO_2_ films, rapid thermal annealing (RTA, UTR‐100, ULTECH) was conducted at 600 °C in an N_2_ environment for 1 minute, resulting in crystallization of m(−122) and m(−111) phases. Both amorphous (as‐deposited) and crystalline (annealed) ZrO_2_ films on TiN substrates were used in subsequent steps for the adsorption of *Inhibitor A* and *Inhibitor B*, followed by the ALD process.

For *Inhibitor A*, carbon tetrafluoride (CF_4_) and sulfur hexafluoride (SF_6_) gases were used. The surface treatment procedures using these gases are illustrated in Figure S11 (Supporting Information). The CF_4_ plasma treatment was conducted on the ZrO_2_ substrate using a reactive ion etching (RIE) system (PERI‐R Series, ULTECH) at a plasma power of 50 W for 1 min, with a CF_4_ gas flow rate of 100 sccm. Following CF_4_ plasma exposure, the substrate underwent thermal annealing at 200 °C for 1 min in a nitrogen atmosphere using the RTA system. For SF_6_ gas treatment, ZrO_2_ substrates were placed in a furnace (SH‐FU‐100^TH^, SAMHEUNG) at temperatures ranging from 200 to 400 °C for 1 min, with an SF_6_ gas flow rate of 50 sccm. The adsorption of *Inhibitor* A onto the ZrO_2_ surfaces was characterized using XPS.

ZrCp(NMe_2_)_3_ molecules were used as precursors for the deposition of ZrO_2_, and as *Inhibitor B* to block Al_2_O_3_ formation. ZrCp(NMe_2_)_3_ molecules as *Inhibitor B* were exposed to the F‐passivated ZrO_2_ surface through Ar carrier gas for 20 s after depositing ZrO_2_. The canister and line temperatures of the ZrCp(NMe_2_)_3_ were maintained the same as that of the ZrO_2_ ALD. The adsorption and removal of *Inhibitor B* were observed using the water contact angle (WCA, Phoenix‐MT(A), SEO) and XPS. Based on previous observations, a discrete feeding method (multiple doses of precursor molecules) for the ZrCp(NMe_2_)_3_ inhibitor on the ZrO_2_ substrate was used for improving the packing density in this study.^[^ ^35^ ^]^ As shown in Figure S12 (Supporting Information), four discrete feedings of ZrCp(NMe_2_)_3_ were the most effective, resulting in WCA values increased and saturated from 33.0° to ≈48.2°. Thus, four doses of ZrCp(NMe_2_)_3_ were set for 20 s each as the optimal conditions. Upon exposing the ZrCp(NMe_2_)_3_‐coated ZrO_2_ surfaces to O_3_ (180 sccm of O_3_ for 60 s), it was observed that the WCA values (33.2°) were the same (33.0°) as that on the ZrO_2_ surface without the ZrCp(NMe_2_)_3_ coating, indicating that the coated ZrCp(NMe_2_)_3_ ligands were either removed or had become reactive. *Inhibitor* removal by O_3_ was also identified using XPS, as the depth profiles of the ZrO_2_/Al_2_O_3_/ZrO_2_ films showed no detectable carbon impurities (Figure S6, Supporting Information).

DFT^[^ ^36^, ^37^ ^]^ calculations for the molecular models were performed using the B3LYP functional,^[^ ^38^, ^39^ ^]^ as implemented in the Gaussian16 package.^[^ ^40^ ^]^ The LanL2DZ basis set^[^ ^41^ ^]^ was employed for the Zr atoms, and the 6–31G(d,p)^[^ ^42^, ^43^, ^44^, ^45^ ^]^ basis set was used for the N, C, and H atoms. In addition to the molecular model calculations, periodic DFT calculations were performed using a slab geometry to represent the amorphous ZrO_2_ surface. To minimize computational cost, molecular models were initially employed rather than surface models to calculate adsorption energies. This choice was justified by the critical role of molecular binding energies in determining the overall results. Selected adsorption energies from the molecular models were later compared to those calculated using surface models of amorphous ZrO_2_. The results showed reasonable agreement, with a deviation of less than 0.3 eV (22%) between the two approaches. The total energies of the slabs and isolated molecules were computed using DFT with the projector‐augmented wave method^[^ ^46^ ^]^ and the Perdew–Burke–Ernzerhof exchange‐correlation functional,^[^ ^47^ ^]^ as implemented in the Vienna Ab initio Simulation Package (VASP).^[^ ^48^, ^49^ ^]^ The wave functions were expanded using plane waves with energy cutoffs up to 500 eV. Owing to the large unit‐cell size of amorphous ZrO_2_, single *k*‐point sampling was used for Brillouin zone integration.

A computational MC simulation was conducted using R software (ver. 3.6.1). The (111) phase was used as the substrate model. The sizes of the molecules were assumed by considering the volumetric sizes obtained from the van der Waals (VDW) radius. It was assumed that the adsorption probability was 100%, and the desorption and physisorption of precursors and intermolecular attractions were ignored. In the MC simulations, the adsorption of each precursor onto a clean surface was simulated separately, displaying the occupied and unoccupied sites on the surface. For instance, a scenario was considered for CpZr(NMe_2_)_3_: based on the DFT analysis, the case where two ligands were lost predominated, representing |−O−Zr(NMe_2_)(Cp).^[^ ^13^ ^]^ In the case of AlMe_3_ adsorption, DFT calculations considered a single CH_3_ ligand exchange forming |−O−Al(CH_3_)_2_). For a monomer AlMe_2_iPrO molecule, DFT calculations considered a single CH_3_ ligand exchange forming |−O−Al(CH_3_)(O(CH_3_)_2_)). In the case of dimer AlMe_2_iPrO, DFT calculations considered the exchange of two CH_3_ ligands at the center between the core Al elements forming |−O−Al(CH_3_)(O(CH_3_)_2_)).^[^ ^15^ ^]^

The MC algorithm involved the following steps: (a) the stage where the Zr precursor existed in the gaseous phase, (b) the stage where the Zr precursor in the gaseous phase collided with the surface, and (c) the stage in which the ligands of the Al precursors (AlMe_3_ and AlMe_2_iPrO) reacted with the surface, leading to the precursor being adsorbed onto the surface. The algorithm proceeded sequentially as follows: (1) the angles and positions of 10,000 Zr precursors were randomly sampled in the gaseous phase; (2) Zr precursors collided with the substrate; (3‐1) if not blocked by previously adsorbed Zr precursors, the gaseous Zr precursors were adsorbed on the surface and moved to stage 4; (3‐2) if already adsorbed precursors blocked adsorption, Zr precursor deposition was prevented; (4‐1) precursor molecules were adsorbed on the surface; (4‐2) If steric hindrance occurred between neighboring precursors, considering the VDW radius, deposition did not occur; (5) angles and positions of 10,000 Al precursors were randomly sampled in the gaseous phase; (6) Al precursors collided with the substrate; (7‐1) if not blocked by previously adsorbed Zr or Al precursors, Al precursors were adsorbed onto the surface and moved to stage 8, (7‐2) if the precursors were already adsorbed, Al precursor deposition was prevented; (8‐1) Al precursor molecules were adsorbed on the surface; and (8‐2) if steric hindrance occurred between neighboring precursors, considering the VDW radius, deposition did not occur. The simulations were conducted 50 times for all cases and included the error range for the surface coverage of the precursors. This methodology was not limited to this study and could be applied to other precursors/molecules to study the changes in surface coverage based on various molecular structures, such as the deposition of multicomponent systems.

The chemical compositions of the deposited films and oxidation states of each element were analyzed using XPS because the surfaces were measured without pretreatment or etching. Topographical images were obtained by TEM (Themis Z, Thermo Fisher Scientific) at an acceleration voltage of 200 kV. For the TEM analysis, ZrO_2_ was deposited on a 15 nm SiN_x_ membrane designed for a TEM grid (TED PELLA, INC.) using 270 cycles to achieve a thickness of ∼30 nm. In the subsequent deposition, the exposure process of either *Inhibitor A* or *Inhibitor B* molecules was performed based on the specific objective. The amount and distribution of each element were identified using EDS and EELS during TEM measurements. To quantify the spatial relationship between Al deposition and GBs, the distance from the location of the highest EDS and EELS Al signal intensity to the nearest GB was measured across multiple line profiles. For each TEM image, more than five line scans were drawn across different GBs within a single grain to ensure comprehensive measurements.

The fabrication procedure of the MIM capacitor is illustrated in Figure S13 (Supporting Information). A 3‐nmthick layer of ALD ZrO_2_ films was deposited on TiN. The ZrO_2_ surfaces both annealed and unannealed, were then exposed to the *Inhibitor A* and *Inhibitor B*. Two cycles of Al_2_O_3_ ALD were performed, followed by the removal of *Inhibitor B* through O_3_ treatment. Subsequently, an additional 3 nm layer of ZrO_2_ was deposited directly in the same chamber. Post‐deposition annealing (PDA) was conducted using performing RTA at 600 °C in an N_2_ environment for 1 min. Subsequently, a 100‐nm‐thick Al was deposited as a metal gate through a patterned shadow mask using an e‐beam evaporator (KVE‐400, Korea Vacuum Tech). Electrical properties based on the capacitance–voltage (*C–V*) and current–voltage (*I–V*) characteristics were determined using a Keysight B1500A semiconductor device parameter analyzer. The electrical properties, such as leakage current and dielectric constant (k value), were measured across multiple devices for consistency and reliability. A minimum of 10 measurements per sample were collected, and the results were presented as mean values. Outliers were carefully identified and removed using the 1.5 interquartile range (IQR) method, ensuring accurate representation of the data trends. All data analysis and visualization were performed using OriginPro 2022.

## Conflict of Interest

The authors declare no conflict of interest.

## Supporting information



Supporting Information

## Data Availability

Research data are not shared.
