# Supplementary material for: Area‐Selective Atomic Layer Deposition on Homogeneous Substrate for Next‐Generation Electronic Devices
Source: Adv Sci (Weinh). 2025 Apr 3;12(22):2414483. doi: 10.1002/advs.202414483 (PMC12165030; doi:10.1002/advs.202414483)
Supplement: Supplementary file 1 — Supporting Information [file ADVS-12-2414483-s001.docx]

**Area-Selective Atomic Layer Deposition on Homogeneous Substrate for Next-Generation Electronic Devices**

Min-Jeong Rhee^a,#^, Byoungjun Won^a,#^, Young-Jin Lim^a,#^, Jeong-Gyu Song^b^, Sunghyun Kim^b^, and Il-Kwon Oh^a,c*^

*^a^ Department of Intelligence Semiconductor Engineering, Ajou University, Suwon, 16499 Republic of Korea*

*^b^ Device Research Center, Samsung Advanced Institute of Technology, Samsung Electronics, 130 Samsung-ro, Suwon, Gyeonggi-do 16678, Republic of Korea*

*^c^ Department of Electrical and Computer Engineering, Ajou University, Suwon, 16499 Republic of Korea*

^*^ Corresponding author’s email: [ikoh@ajou.ac.kr](mailto:ikoh@ajou.ac.kr) ^#^ These authors are equally contributed.


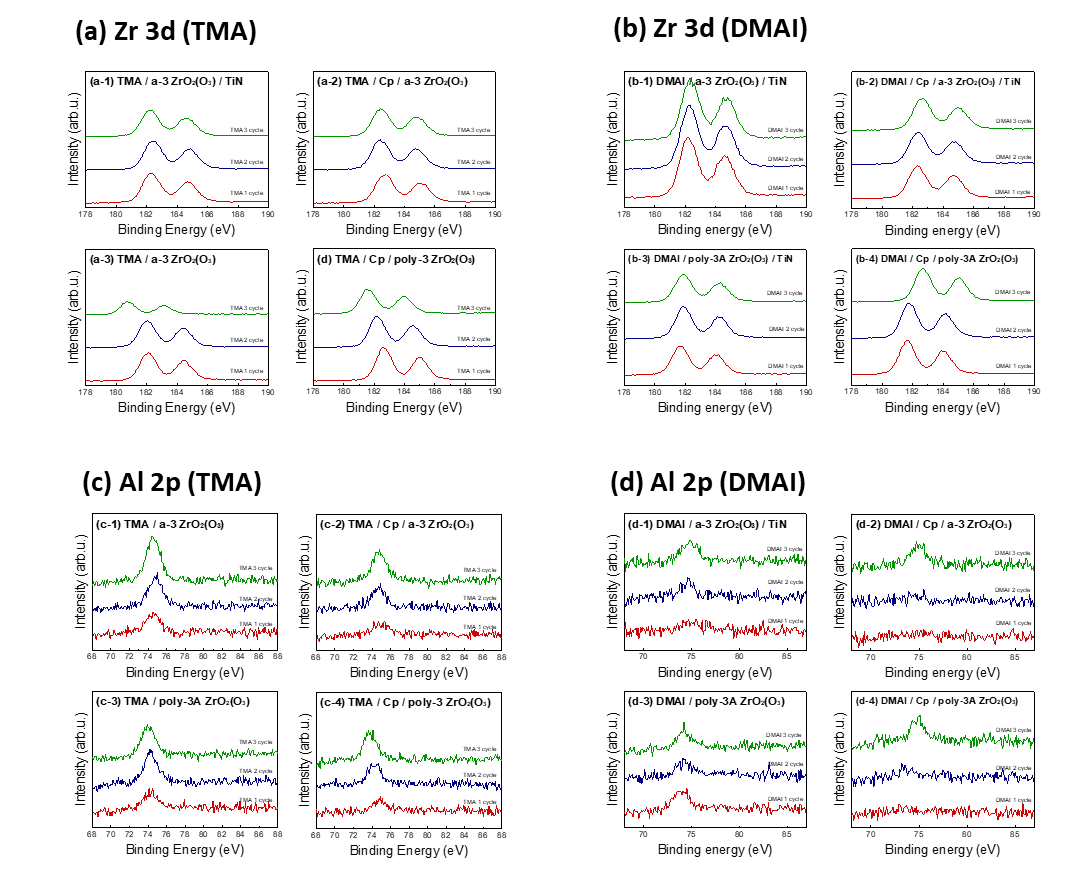


**Figure S1** XPS core-level spectra of (a) XPS core-level spectra showing Zr 3d levels and (b) Al 2p levels for Al_2_O_3_ deposited on ZrO_2_ using TMA as the Al precursor. (c) Zr 3d levels and (d) Al 2p levels for Al_2_O_3_ deposited on ZrO_2_ using DMAI as the Al precursor. The spectra are shown for varying numbers of ALD cycles, all in the presence of inhibitor B


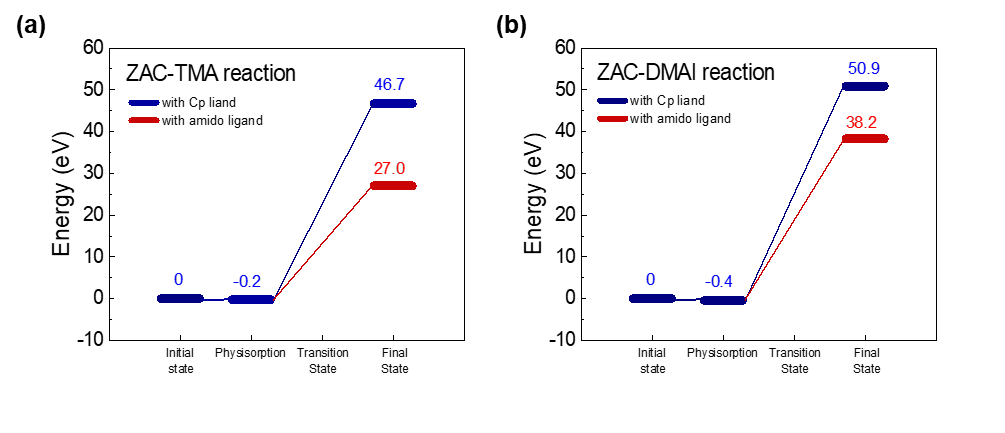


**Figure S2** Reaction energies with (a) ZAC-TMA reaction and (b) ZAC-DMAI reaction


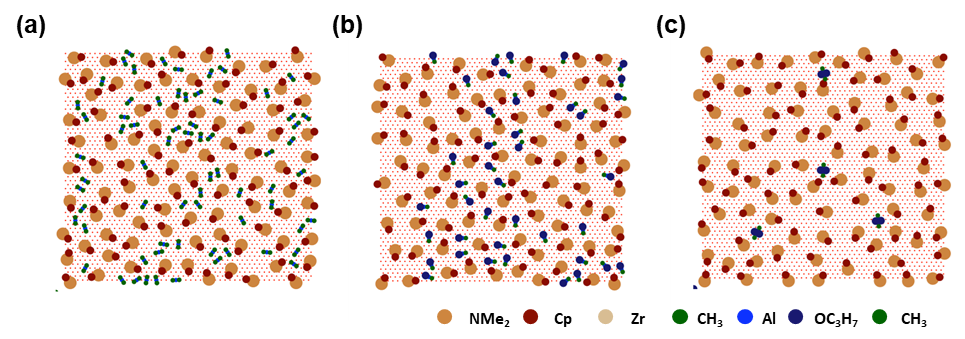


**Figure S3** MC simulation results (a) TMA on CpZr(NMe_2_)_3_ result, (b) DMAI on CpZr(NMe_2_)_3_ result when DMAI acts as a monomer, and (c) DMAI on CpZr(NMe_2_)_3_ result when DMAI acts as a dimer


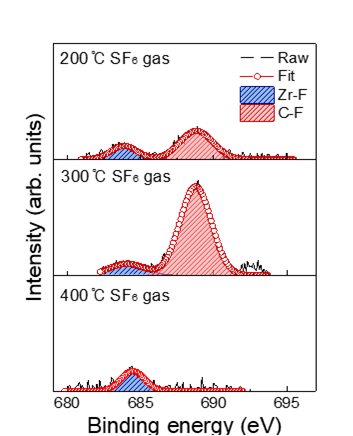


**Figure S4** Chemical composition of ZrO_2_ surface after halogenization (a) F 1s peak in the XPS data of SF_6_ gas trap on ZrO_2_ 10 nm at 200 ℃, 300 ℃, and 400 ℃

**Figure S5** Schematic illustration of the preparation process for 1) ZAZ, 2) ZAZ+Cp, 3) ZAZ+F, and 4)ZAZ+F+Cp devices


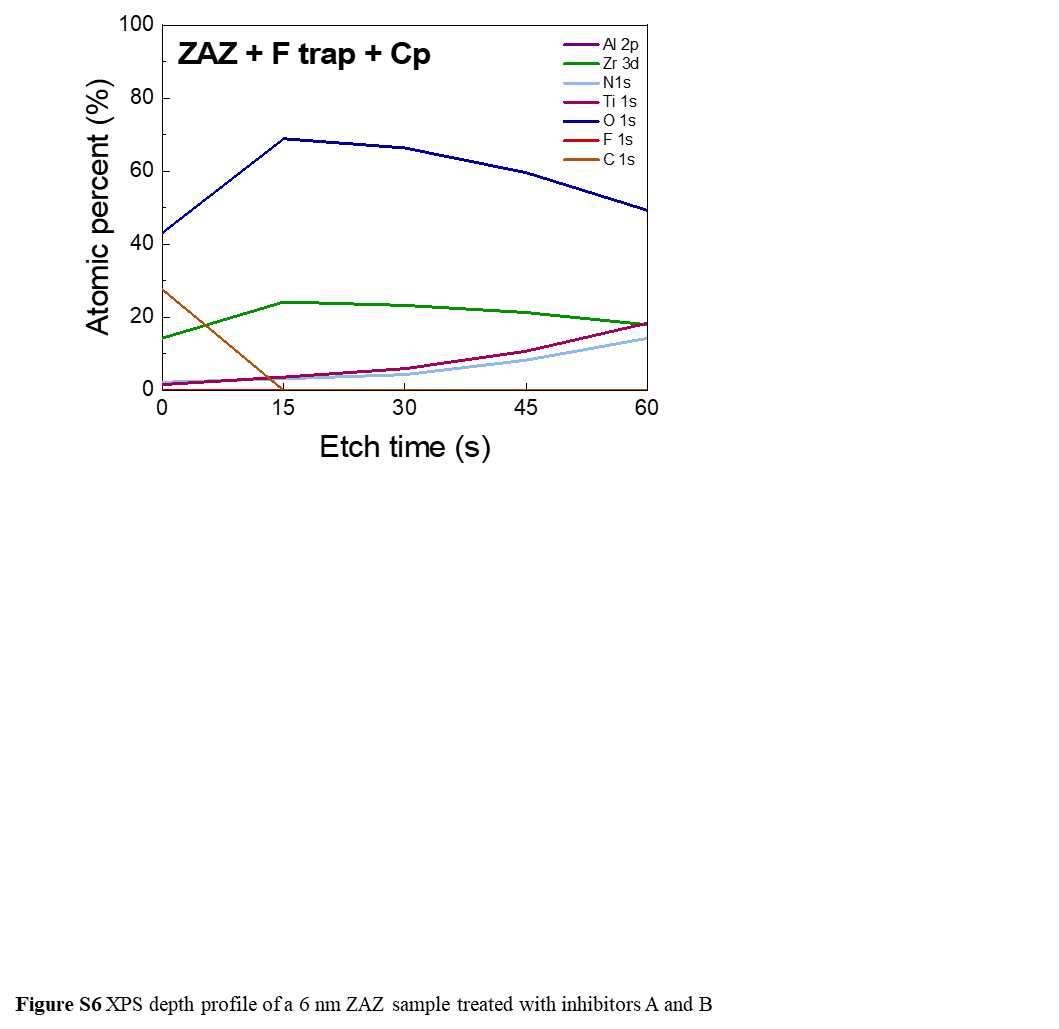


**Figure S6** XPS depth profile of a 6 nm ZAZ sample treated with inhibitors A and B


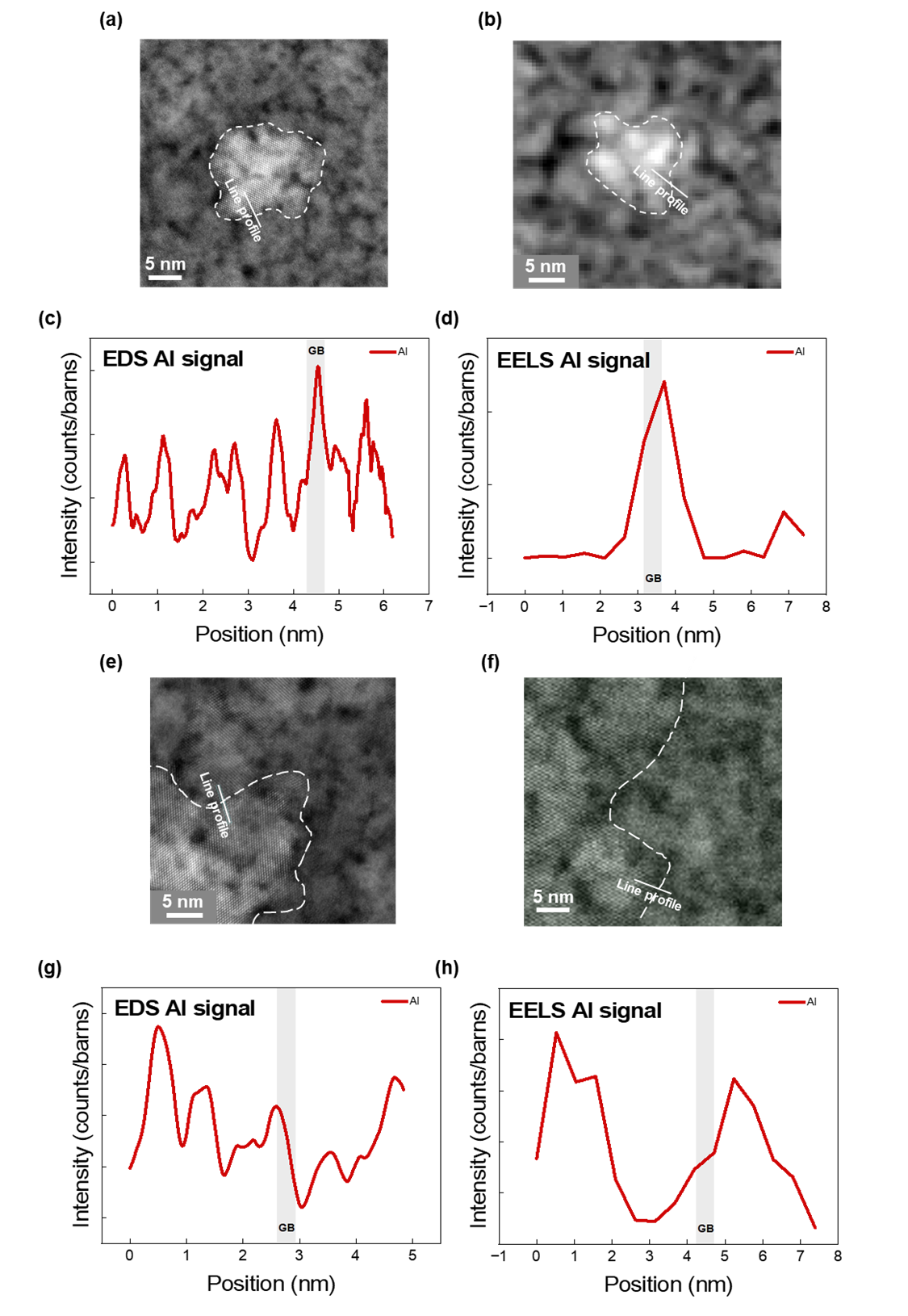


**Figure S7** (a) TEM image with a line profile corresponding to the EDS results in (c) for the AS-ALD sample. (b) TEM image with a line profile corresponding to the EELS results in (d) for the AS-ALD sample. (c) EDS line profile showing the Al peak near the GB of (a). (d) EDS line profile showing the Al peak near the GB of (b). (e) TEM image with a line profile corresponding to the EDS results in (g) for the general ALD comparison sample. (f) TEM image with a line profile corresponding to the EDS results in (h) for the general ALD comparison sample. (g) EDS line profile showing the Al peak near the GB of (e). (h) EDS line profile showing the Al peak near the GB of (f).

**
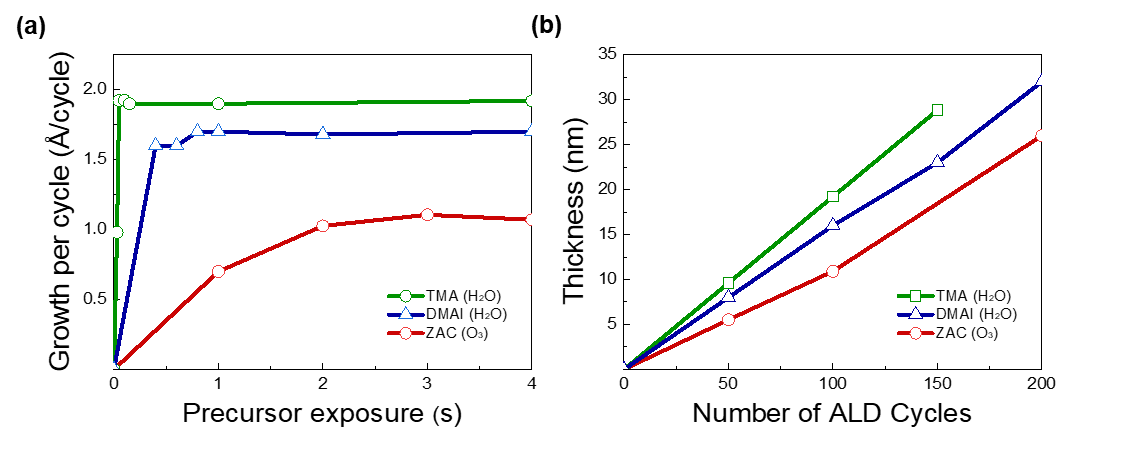
 Figure S8** Growth characteristic (a) saturation curve when using TMA (H_2_O), DMAI (H_2_O), and ZAC (O_3_) respectively (b) linearity curve when using TMA (H_2_O), DMAI (H_2_O), and ZAC (O_3_) respectively


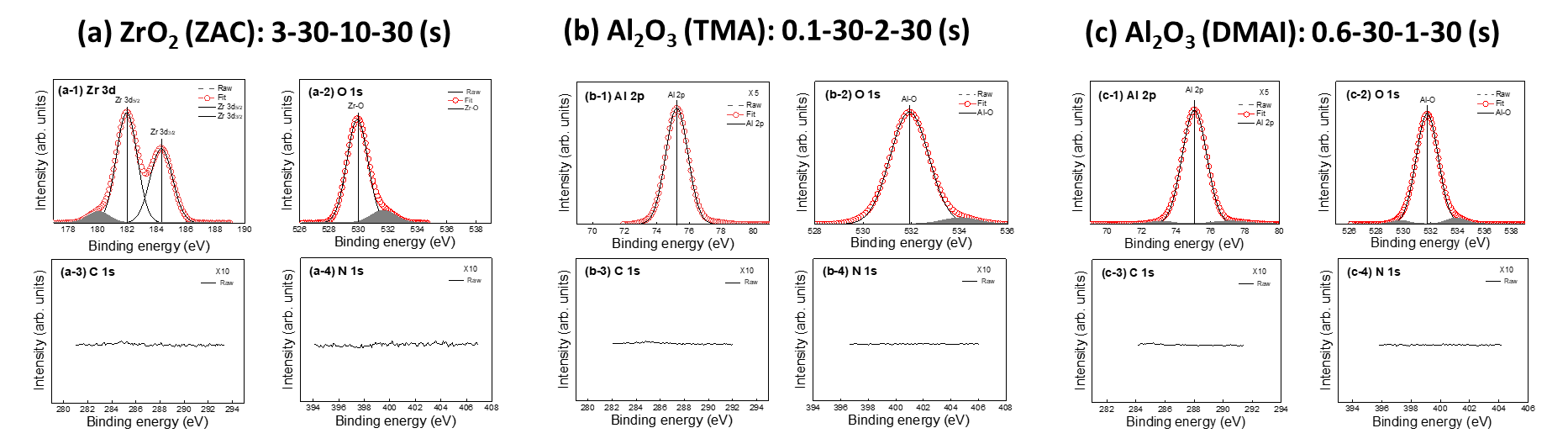


**Figure S9** XPS peaks of (a) ZrO_2_ using ZAC (O_3_), (b) Al_2_O_3_ using TMA (H_2_O), (c) Al_2_O_3_ using DMAI (H_2_O)

|  | Zr 3d | Al 2p | O 1s | C 1s | N 1s |
| --- | --- | --- | --- | --- | --- |
| ZAC (O_3_) | 31.3 | - | 68.8 | < 1 | < 1 |
| TMA (H_2_O) | - | 41.6 | 58.1 | < 1 | < 1 |
| DMAI (H_2_O) | - | 41.8 | 58.1 | < 1 | < 1 |

**Table S1** Chemical composition of ZrO_2_ using ZAC (O_3_), Al_2_O_3_ using TMA (H_2_O), Al_2_O_3_ using DMAI (H_2_O)


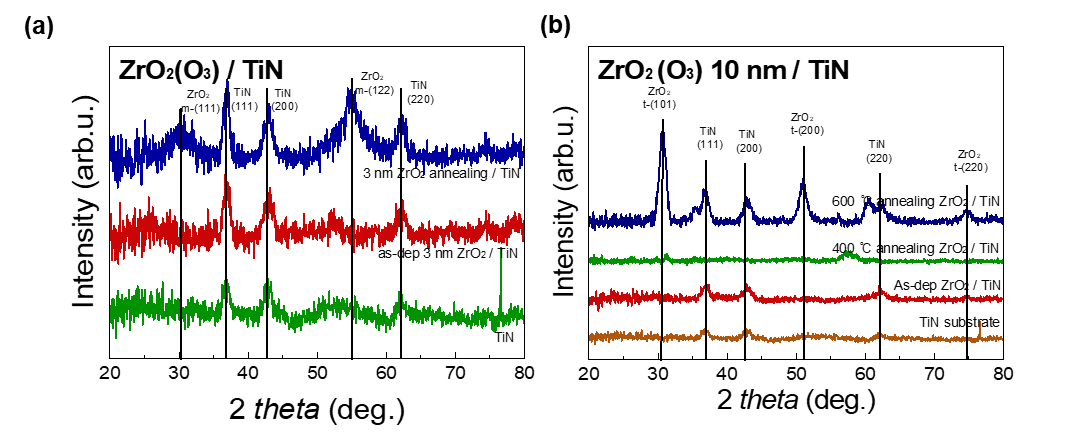


**Figure S10** Crystallinity of (a) 3nm-ZrO_2_ (b) 10 nm-ZrO_2_. The grain size for the t-(101) plane is approximately 2.6 nm for the 3 nm-ZrO_2_ film and 7.6 nm for the 10 nm-ZrO_2_ film.


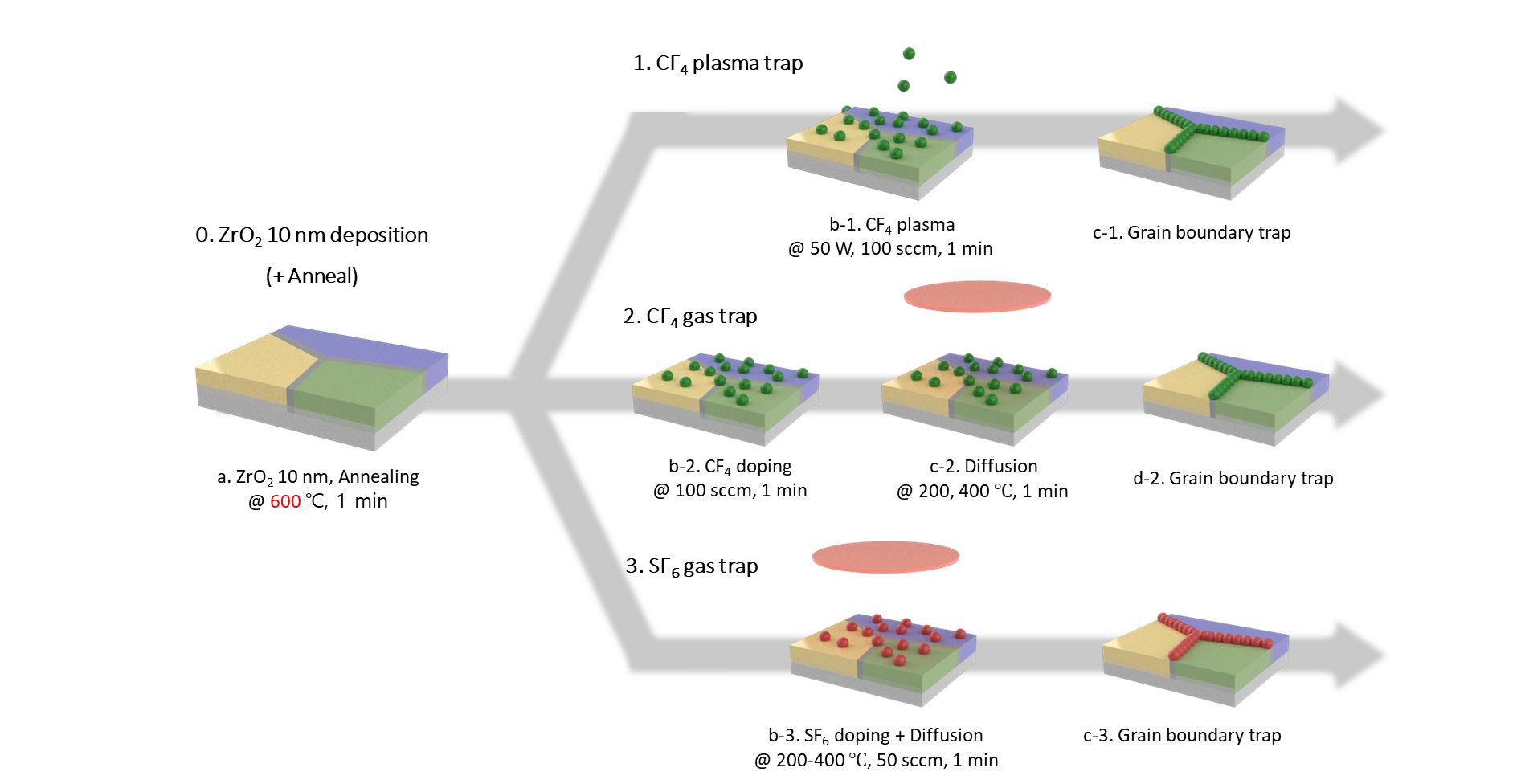


**Figure S11** Schematic illustration of the preparation process for F-treated samples


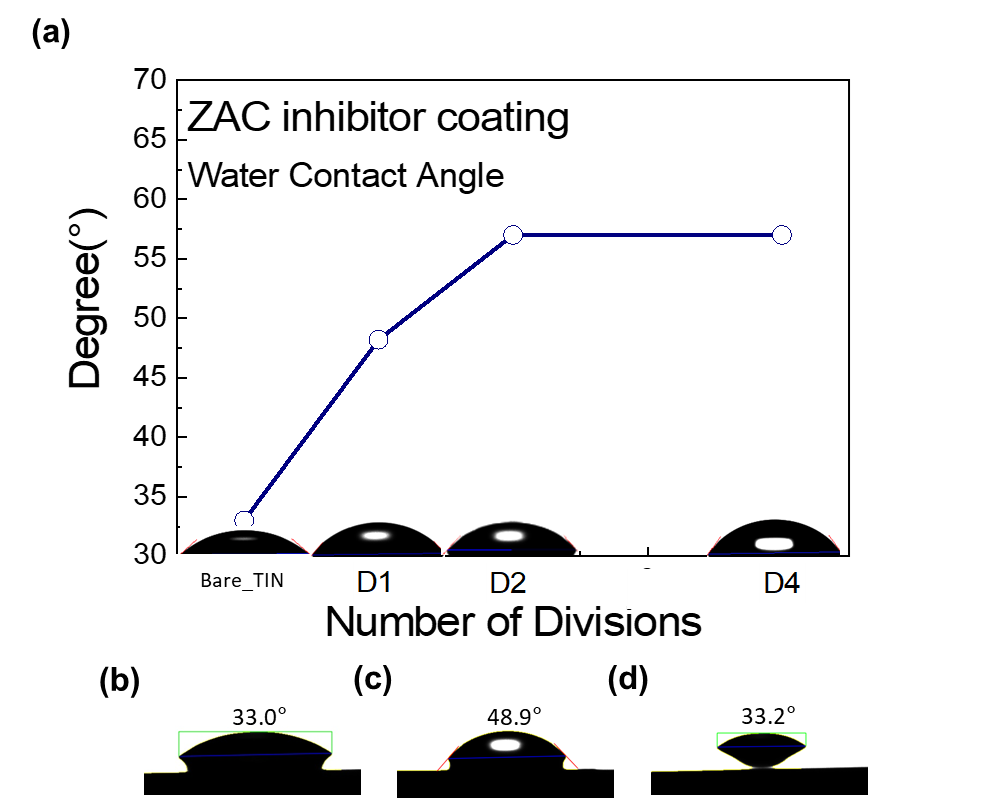


**Figure S12** (a) Water contact angle measurements showing the effect of discrete ZAC feedings on ZrO_2_ surfaces, water contact angle comparing (b) bare ZrO_2_ (3 nm) with a WCA of 33.0°, (c) ZrO_2_ after treatment with Inhibitor B, showing an increased WCA of approximately 48.2°, and (d) ZrO_2_ after subsequent O_3_ treatment for 60 seconds, where the WCA returns to 33.2°


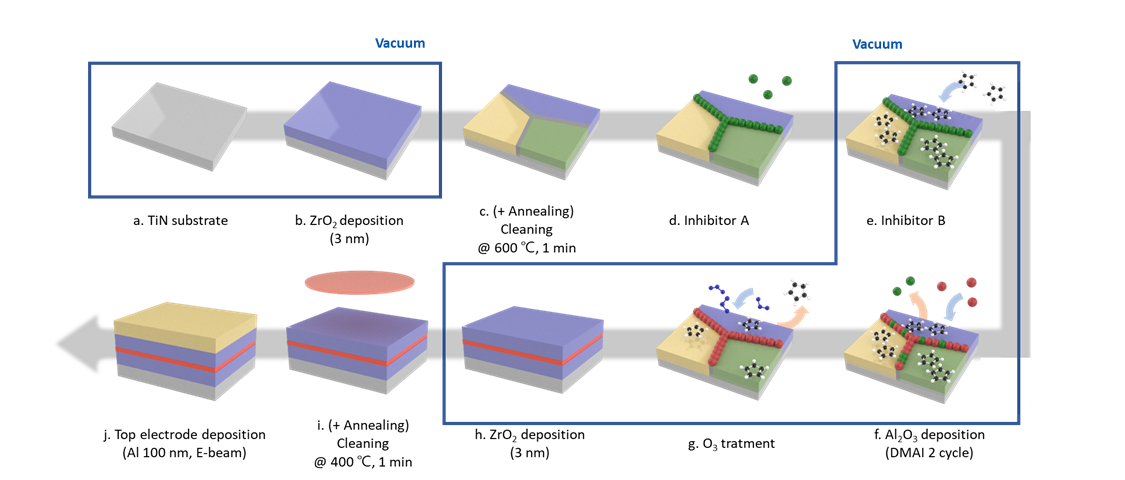


**Figure S13** Schematic illustration of the fabrication procedure for the Metal-Insulator-Metal (MIM) capacitor
